# Memory reactivation generates new, adaptive behaviours that reach beyond direct experience

**DOI:** 10.1038/s41598-024-78906-1

**Published:** 2024-12-03

**Authors:** Annalise B. Rawson, Sumedha Nalluru, Jill X. O’Reilly, Helen C. Barron

**Affiliations:** 1 Wellcome Centre for Integrative Neuroimaging, University of Oxford, FMRIB, John Radcliffe Hospital, Oxford, UK; 2 Medical Research Council Brain Network Dynamics Unit, Nuffield Department for Clinical Neurosciences, University of Oxford, Oxford, UK; 3 Department of Experimental Psychology, University of Oxford, Oxford, UK

**Keywords:** Consolidation, Hippocampus, Cognitive neuroscience

## Abstract

Periods of rest and sleep help us find hidden solutions to new problems and infer unobserved relationships between discrete events. However, the mechanisms that formulate these new, adaptive behavioural strategies remain unclear. One possibility is that memory reactivation during periods of rest and sleep has the capacity to generate new knowledge that extends beyond direct experience. Here, we test this hypothesis using a pre-registered study design that includes a rich behavioural paradigm in humans. We use contextual Targeted Memory Reactivation (TMR) to causally manipulate memory reactivation during awake rest. We demonstrate that TMR during rest enhances performance on associative memory tests, with improved discovery of new, non-directly trained associations, and no change observed for directly trained associations. Our findings suggest that memory reactivation during awake rest plays a critical role in extracting new, unobserved associations to support adaptive behavioural strategies such as inference.

**Supplementary Information:**

The online version contains supplementary material available at 10.1038/s41598-024-78906-1.

## Introduction

Periods of sleep and awake rest are reported to aid our ability to make inferences, perform abstraction and acquire insight^1,2^. For example, periods of rest/sleep appear to help us find hidden solutions to new problems^3,4^, infer unobserved relationships between discrete events^5,6^, and, anecdotally, may allow pivotal insight that can be used to guide scientific discovery^7^. Critically, these cognitive processes underpin behavioural flexibility, by providing a means to generate new, adaptive behavioural strategies, without the need for explicit learning or feedback. However, the mechanisms that formulate new, adaptive behavioural strategies during periods of sleep and awake rest remain unclear.

One possibility is that during rest/sleep, memory processing provides an opportunity to formulate previously unobserved relationships by restructuring and integrating learned information. At the neural level, this restructuring and integration of learned information may occur during memory reactivation, where information encoded during prior wakefulness is ‘replayed’ during periods of rest and sleep^8–10^. At the cellular level, ‘replay’ may be characterised by sequences of spiking activity that recapitulate previous waking experience on a temporally compressed scale. Evidence in rodents demonstrates a causal role for memory reactivation in consolidating or strengthening newly learned information^11–14^. However, a number of recent studies suggest that memory reactivation can go beyond veridical replay of past experience. For example, during periods of rest/sleep, spiking activity in the hippocampus can depict spatial trajectories that have never been directly explored^15–17^, and pair sensory cues that have not been experienced together but may be indirectly related^6^. Together these studies suggest that hippocampal spiking activity during periods of rest/sleep has capacity to generate novel sequences, that deviate from past experience. However, it remains unclear whether these physiological phenomena translate into new adaptive *behaviours*, such as our ability to make inferences, perform abstraction and acquire insight. To address this knowledge gap, here we ask: does memory reactivation facilitate new, adaptive behavioural strategies?

To answer this question, we conducted a pre-registered study to obtain a rich readout of the behavioural consequences of memory reactivation. While studies in rodents benefit from allowing memory reactivation to be measured and manipulated at the cellular level, the behavioural complexity of tasks is typically limited. Here, to investigate the effect of memory reactivation on adaptive behaviour we designed a new behavioural paradigm in humans. Our experimental design allowed for: *(1)* a rapid learning paradigm; *(2)* causal manipulation of memory reactivation during periods of awake rest; *(3)* rich exploration of the behavioural consequences of memory reactivation.

To investigate the causal relationship between memory reactivation and behavioural performance, we used targeted memory reactivation (TMR). TMR involves pairing sensory cues with specific memories during learning, before re-exposing participants to these cues during offline periods of sleep/rest^18–22^. This re-exposure has been shown to bias the content of hippocampal memory reactivation towards the cued associations^23^, thus providing a causal tool to manipulate memory reactivation. Capitalising on this approach, we designed a novel TMR protocol: contextual auditory TMR. Rather than cueing specific learned stimuli during rest/sleep, we paired each of two learned ‘maps’ *as a whole* with a contextual auditory background music track (ambient sound), which was then used as the TMR cue. We reasoned that contextual cueing would favour reactivation of the cued map, whilst allowing the sequence of reactivation within the map to take its natural course.

We then coupled contextual TMR with a learning paradigm that allowed us to assess the effects of memory reactivation on behaviour. Specifically, we designed the task to test the effect of memory reactivation on both directly and non-directly trained associations, while also dissociating between associative and contextual aspects of memory, together with participants’ ability to navigate.

We demonstrate that biasing memory reactivation during wakeful rest (using contextual TMR) enhances discovery of new, non-directly trained associations, with no change observed for directly trained associations. We also show that biasing memory reactivation enhances performance on associative memory tests, with no benefit observed for contextual memory tests or participants’ ability to navigate. Together these findings demonstrate that memory reactivation during periods of rest plays a critical role in extracting new, unobserved information to support adaptive behavioural strategies. Moreover, our findings challenge the view that the primary function of memory reactivation during awake rest is to strengthen directly learned information. Rather, these findings suggest that memory reactivation during periods of rest may additionally, or even primarily, facilitate behaviours that extend beyond direct experience to foster adaptive behaviours, such as inference and the discovery of novel associations.

## Results

### Task design and learning performance

**Fig. 1:**
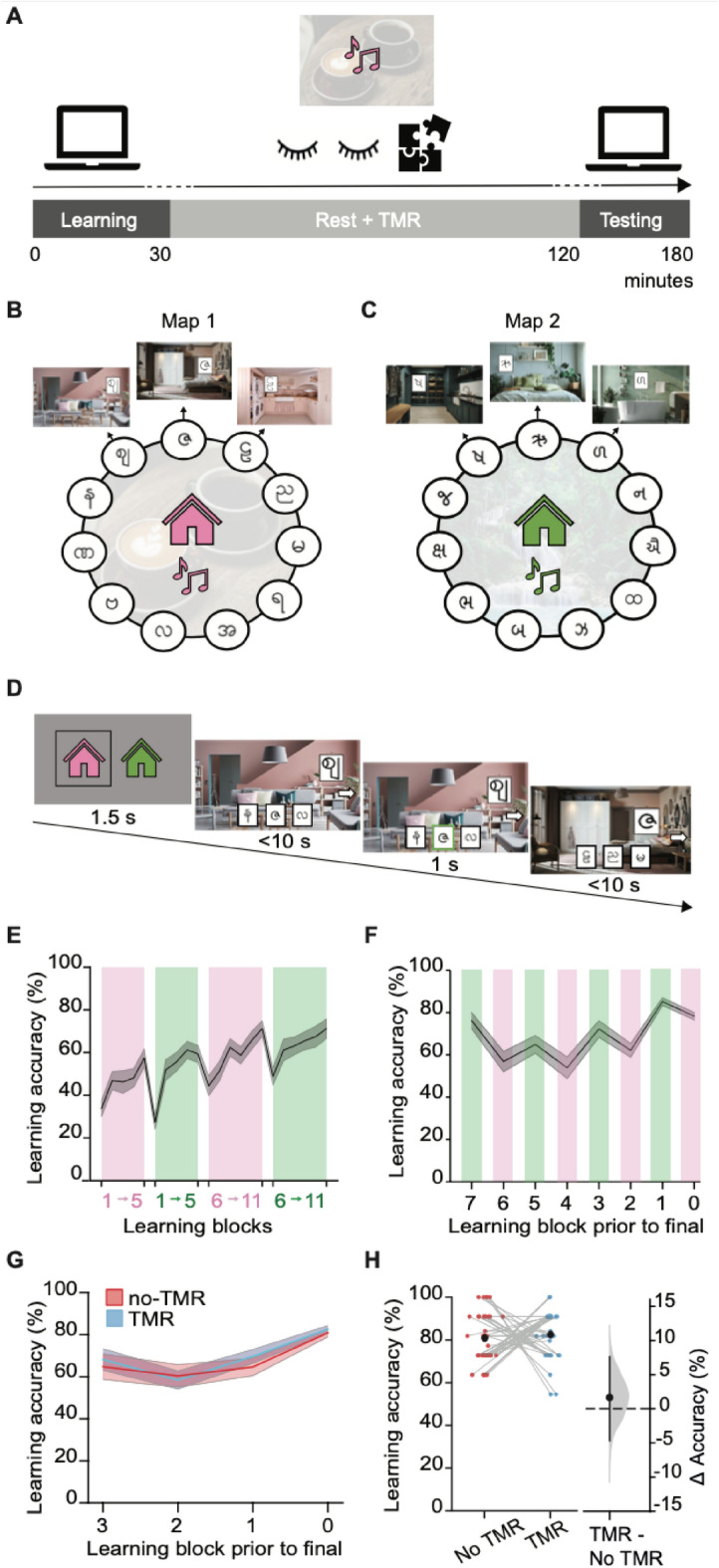
Behavioural task design and learning performance. **(A)** Study timeline. The study was split into the learning phase, rest + TMR phase, and testing phase. During the learning phase, the contextual background music was played during trials on each of the respective maps. During rest + TMR, the contextual music for the TMR map was played to bias memory reactivation towards that map (fully counterbalanced across participants). During the rest + TMR phase, participants alternated between 10 min eyes closed rest and 20 min jigsaw puzzle, for a total of 90 min. After the rest + TMR phase, participants completed the testing phase during which they underwent a series of tests to assess the effect of manipulating memory reactivation using TMR on behaviour. **(B-C)** Task schematic. Participants learned two maps of associations: **(B)** map 1: a pink house; **(C)** map 2: a green house; fully counterbalanced across participants. Each map consisted of 11 nodes with each node (room) containing a unique scene and cue (symbol). During learning, each map was paired with a different contextual background music track (café or jungle music; fully counterbalanced across participants). **(D)** Example trial during the learning phase. Participants learned associations between rooms (scene + cue) and adjacent cues on the map, via sequences of three-alternative-forced-choice trials, always traversing the map in a clockwise direction. At the start of each block of trials for each map, participants were shown the current house (pink or green). On each trial, participants started at a node with an arrow pointing right. Participants were required to choose the symbol in the adjacent room from three options provided at the bottom of the screen, before moving to the adjacent room and receiving feedback. **(E)** Learning accuracy during first learning session. Participants alternated between blocks of training for each map (example shown by pink/green colours; order counterbalanced across participants). Final mean accuracy: 69.77% (SEM = 1.93%). **(F)** Learning accuracy in second learning session. N = 38 participants did not reach criterion on the first learning session and proceeded to the second learning session, where participants alternated between shorter blocks of training for each map (example shown by pink/green colours; order counterbalanced across participants). Mean accuracy across the final block of learning for all participants: 81.68% (SEM = 0.61%). **(G)** Learning accuracy across the final learning blocks of the second learning session, for maps that subsequently became the TMR map (blue) and the no-TMR map (red). **(H)** In the final learning block there was no significant difference in learning accuracy between maps later assigned to TMR and no-TMR across all participants (n = 40, p = 0.604). Left: raw data points for no-TMR map (red; left) and TMR map (blue; right); each data point is mean accuracy for one participant; black dot, mean; black ticks ± SEM. Right: difference in mean percentage accuracy between no-TMR and TMR maps shown using bootstrap-coupled estimation (DABEST) plots. Effect size for the difference between no-TMR and TMR maps was computed from 10,000 bias-corrected bootstrapped resamples: black dots, mean; black ticks, 95% confidence interval; filled-curve, sampling-error distribution.

To investigate the effect of memory reactivation on subsequent behaviour we designed a map learning task that allowed us to dissociate memory for directly and non-directly trained associations, together with associative, contextual and navigational information. The map learning task was then coupled with a TMR manipulation to bias memory reactivation (Fig. 1A).

First, participants learned two maps of associations (‘learning phase’). Each map included 11 rooms from either a pink house map (map 1, Fig. 1B) or a green house map (map 2, Fig. 1C). The rooms in each map were arranged in a circular graph structure, although participants were not explicitly made aware of this. Importantly, during learning, participants only ever saw trajectories between adjacent rooms (or nodes) on the graph, so the relationships between non-adjacent nodes had to be inferred.

Within each map, each room (or node) consisted of a unique scene and cue (symbol) (Fig. 1B-C). During learning, participants learned room-to-cue associations. On each trial of the learning task participants were shown a room (scene + cue) and asked to identify the cue in the adjacent room, using a three-alternative-forced choice task (Fig. 1D). Adjacent rooms were experienced in sequence, moving in the clockwise direction only. Learning alternated between blocks of sequences for each map (Fig. 1E-F). To allow application of TMR during a period of rest, for each map a different contextual background music track was played throughout learning (map 1 with café music; map 2 with jungle music; fully counterbalanced across participants) (Fig. 1B-C).

Participants continued learning room-to-cue associations until they reached a criterion accuracy of 75%, either at the end of a first learning session, or at the end of shorter sub-blocks completed as part of a second learning session (Fig. 1E-F; see Methods). By the end of the learning task, all participants included in the study reached criterion, achieving a mean accuracy of 81.68% (Fig. 1G). Critically, there was no significant difference in the final learning accuracy between the maps subsequently used for the TMR and no-TMR conditions (p = 0.604) (Fig. 1H).

### Biasing memory reactivation during rest using TMR

After the learning phase, all participants underwent a TMR manipulation. TMR involves pairing sensory stimuli with specific memories during learning, before re-exposing participants to these stimuli during offline periods of sleep/rest. Previous studies demonstrate that TMR can be used to bias the content of hippocampal replay towards those memories that have been linked to the sensory stimuli played during sleep^23^. TMR therefore provides a causal tool to bias the content of memory reactivation during offline periods^18–22^. Here, during a period of awake rest (‘rest + TMR phase’), participants were re-exposed to the contextual background music track linked to one of the two maps (i.e., café or jungle music; Fig. 1A).

While previous TMR protocols have used punctate auditory cues, here we implemented a novel approach by delivering TMR using a contextual auditory manipulation. This contextual TMR protocol was designed to bias reactivation towards an entire memory map, thus providing an opportunity to explore diverse consequences of memory reactivation on subsequent behaviour without interfering with the endogenous replay sequences that might occur during memory reactivation. For example, if memory reactivation strengthens learned associations, *and/or* discovery of new, previously unexperienced associations, then biasing memory reactivation towards the TMR map should enhance behavioural measures of these processes for the TMR relative to the no-TMR map.

### Testing the effect of TMR on memory performance

**Fig. 2:**
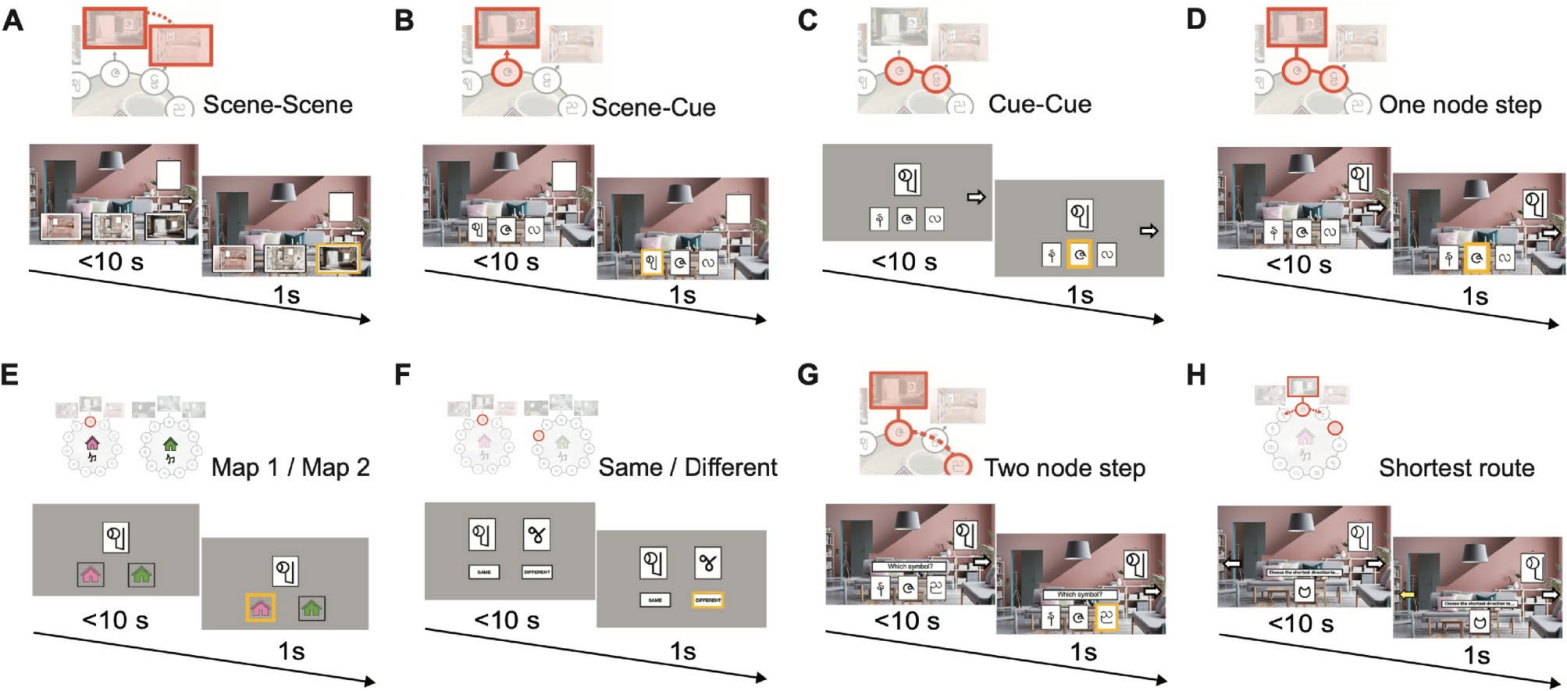
Schematics of the 8 tasks used during the post-rest memory tests. The 8 test tasks (**A**-**G**) are shown in the order in which they were completed by participants. **(A)** Scene-scene: participants were shown a scene and asked to identify the scene in the neighbouring node. **(B)** Scene-cue: participants were shown a scene without its paired cue and asked to identify the missing cue. **(C)** Cue-cue: participants were shown a cue without its paired scene and asked to identify the cue from the neighbouring node. **(D)** One node step: as in the learning task, participants were shown a scene with its paired cue and asked to identify the cue from the neighbouring node. **(E)** Map 1/map 2: participants were shown a cue and asked whether it belongs to the pink or green house. **(F)** Same/different: participants were shown two cues and asked if they belong to the same map or different maps. **(G)** Two node steps: participants were shown a room (scene + cue) and were asked to identify the cue *two* node steps to the right. **(H)** Shortest route: while trial sequences in the learning phase were delivered in a clockwise direction only, here participants were shown a room (scene + cue) and were required to indicate the shortest route to a probe cue, positioned two nodes away, by selecting either clockwise (right) or anticlockwise (left) directed travel around the underlying map. This task was designed to assess participants’ ability to abstract out the underlying ring structure of the memory maps, before using this knowledge to execute efficient navigation.

**Fig. 3:**
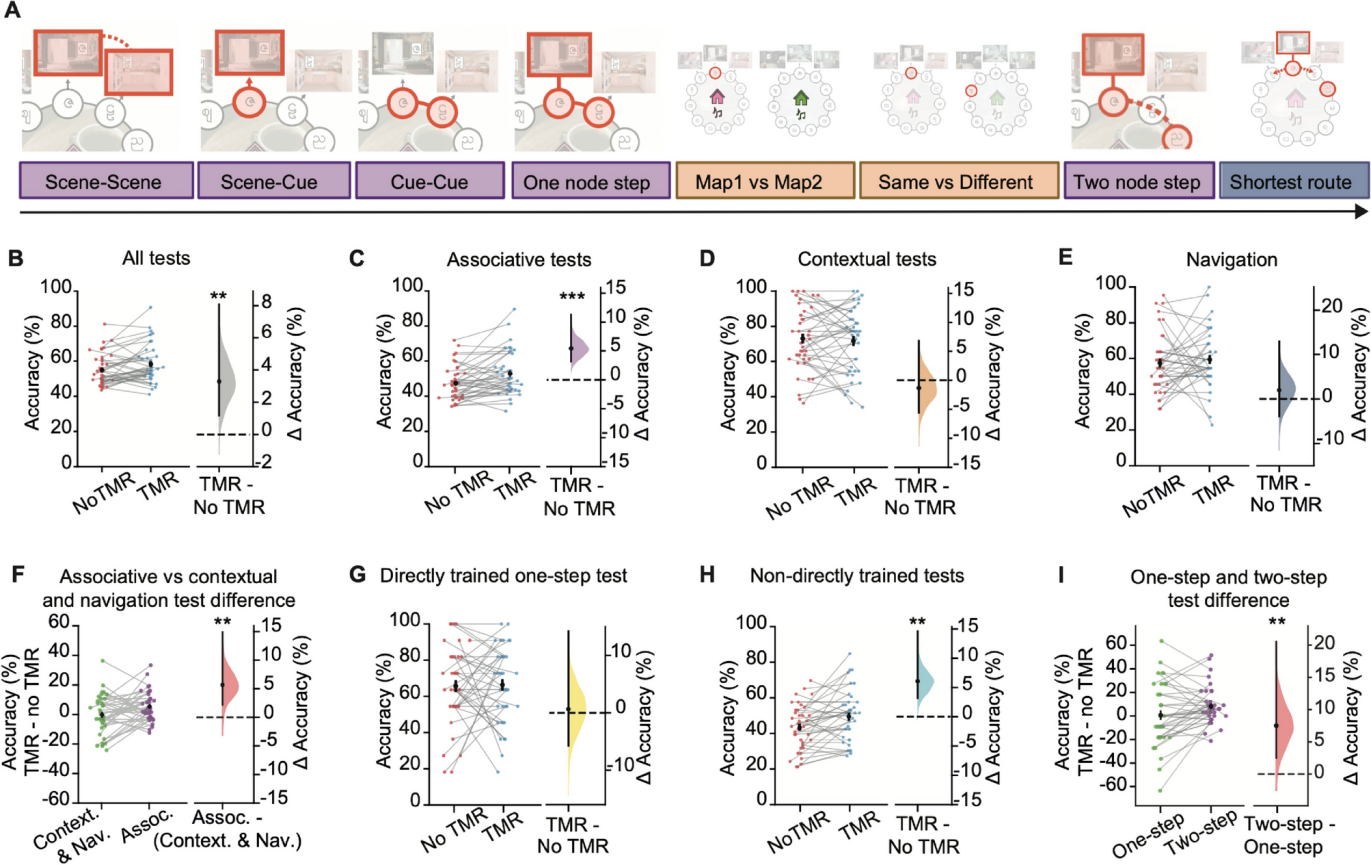
Performance on post-rest memory tests reveals TMR effect for non-directly trained associative tests. (**A**) Schematic showing battery of tests included in the test phase to assess the effect of TMR on different aspects of memory. Associative memory tests shown in purple (scene-scene, scene-cue, cue-cue, one node step, and two node step inference) test knowledge of within-map associations. Contextual memory tests shown in orange (map1/map2, same/different) test between-map knowledge. The navigation memory test shown in blue-grey (shortest route) tests participants’ ability to navigate the underlying memory maps. **(B-I )** Left: raw data points for no-TMR group (red; left) and TMR group (blue; right) **(B-E, G-H)**, or for two different types of task (green, left; purple, right)** (F, I)**. Each data point is mean accuracy for one participant; black dot, mean; black ticks ± SEM. Right: difference in mean percentage accuracy between no-TMR and TMR groups **(B-E, G-H)** or between two different types of task **(F, I)** shown using bootstrap-coupled estimation (DABEST) plots. Effect size for the difference between no-TMR and TMR groups was computed from 10,000 bias-corrected bootstrapped resamples: black dots, mean; black ticks, one-tailed 95% confidence interval; filled-curve, sampling-error distribution. **(B)** TMR had a significant effect on average memory performance across all tests (p = 0.007). **(C-E)** TMR had a significant effect on associative test performance (***C***, p < 0.001), with no effect observed for contextual tests (***D***, p = 0.696) or for the navigation test (***E***, p = 0.284). **(F)** The effect of TMR on associative tests was significantly greater than that observed for contextual and navigation tests (***F***, p = 0.007). **(G, H)** No significant effect of TMR was observed for directly trained tests (***G***, p = 0.426; one node step), while a significant effect of TMR was observed for all non-directly trained tests (***H***, p < 0.001; scene-scene, scene-cue, cue-cue, two-step). **(I)** The effect of TMR on the indirect two node step test was significantly greater than that observed on the direct one node step test (p = 0.010).

To investigate the effect of memory reactivation on subsequent behaviour, after the rest + TMR phase participants performed a battery of 8 pre-registered tests. Each of the 8 tests were designed to target a different aspect of memory (‘test phase’, Figs. 1A, 2, 3A). During the test phase, the auditory contextual background cues (café or jungle music) were not played, and participants were not given immediate feedback on their responses.

The 8 tests were designed to test associative, contextual and map knowledge, by testing ***within-map*** associations (‘associative tests’), participants’ ability to delineate ***between-maps*** (‘contextual tests’), and participants’ ability to abstract out the underlying ring structure to navigate around the maps (‘navigation test’).

The associative tests included 5 different tests, designed to test participants’ memory for ***within-map*** associations, between: (1) scenes across neighbouring nodes (‘*scene-scene*’, Fig. 2A); (2) scene and cue within node (‘*scene-cue*’, Fig. 2B); (3) cues across neighbouring nodes (‘*cue-cue*’, Fig. 2C); (4–5) node (scene + cue) to cue information across neighbouring nodes (*4*, ‘*one node step*’, Fig. 2D), and across nodes that are two-steps away on the map (*5*, ‘*two node steps*’, Fig. 2G). One of these associative tests, namely ‘*one node step’ *(Fig. 2D), had the same structure as the trials implemented during the learning phase, thus serving as a test for knowledge of directly trained associations. The remaining four associative tests had a different structure to trials used during the learning phase and tested knowledge of associations that were not directly trained.

In addition to these 5 associative tests used to assess associative memory, in the test phase we included two tests to assess participants’ ability to delineate ***between*** the two maps (‘contextual tests’). In the first of the two contextual tests participants were required to indicate which of the two possible maps a cue belonged to (‘map1/map2’, Fig. 2E). In the second of the two contextual tests, participants were required to indicate whether two cues came from the same map or not (‘same/different’, Fig. 2F). Finally, there was a navigation test (‘shortest route’), which tested participants’ ability to navigate efficiently around each map in a bi-directional manner (Fig. 2H), despite having learned each map in uni-directional (clockwise) direction. Together with our TMR manipulation, this battery of 8 tests provided an opportunity to assess whether associative memory, contextual knowledge, or participants’ ability to navigate, is affected by memory reactivation, both for directly trained and non-directly trained information.

### TMR improves associative memory, but not contextual memory or participants’ ability to navigate

To establish how memory reactivation affects subsequent behaviour, we first assessed participants’ performance on the TMR vs no-TMR map across all 8 tests. Consistent with our pre-registered predictions, participants performed significantly better on the TMR map compared to the no-TMR map (TMR vs no-TMR map: p = 0.007, Fig. 3B), although no significant differences were observed in reaction time data (Supplementary Fig. 2–3). This shows that using TMR to evoke memory reactivation during awake rest enhances overall memory performance for the TMR map.

To establish whether TMR facilitates associative or contextual memory, or our ability to navigate, we next assessed performance on the different types of tests. For associative tests, which tested knowledge for associations within a map (Fig. 2A-D, G), participants performed significantly better on the TMR compared to the no-TMR map (p < 0.001, Fig. 3C). For contextual tests, which tested knowledge of which rooms and symbols belonged on which map (Fig. 2E-F), no significant difference was observed between the two maps (p = 0.696, Fig. 3D). For the navigation test, which tested participants’ ability to navigate around each of the two maps (Fig. 2H), no significant difference was observed between the two maps (p = 0.255, Fig. 3E). Moreover, the TMR effect observed for associative tests was significantly greater than the TMR effect for contextual and navigational memory tests (p = 0.007, Fig. 3F). This suggests that manipulating memory reactivation using awake contextual TMR benefits associative memory, but not contextual memory or participants’ ability to efficiently navigate the underlying maps.

### TMR enhances knowledge of non-directly trained associations

Next, as specified in our pre-registration, across associative tests we asked whether biasing memory reactivation using TMR strengthens knowledge for learned information and/or generates new knowledge that deviates from direct experience. To this end, we divided the 5 associative tests in the testing phase into those testing knowledge for associations that were ‘directly’ and ‘non-directly’ trained. Only the one node step test (Fig. 2D), was equivalent to the initial learning task (but now with shuffled trial order) and therefore classified as ***directly trained***. All other associative tests, namely scene-scene, scene-cue, cue-cue and two node step were different from the learning task and therefore classified as ***non-directly trained***. More specifically, participants were never explicitly trained on: which room came next in the sequence (scene-scene); which cue belonged in each room (scene-cue); cue associations in the absence of the background scenes (cue-cue), or inference across two or more nodes in a map (two node step). For both directly and non-directly trained tests we compared performance on the TMR and no-TMR map.

**Fig. 4:**
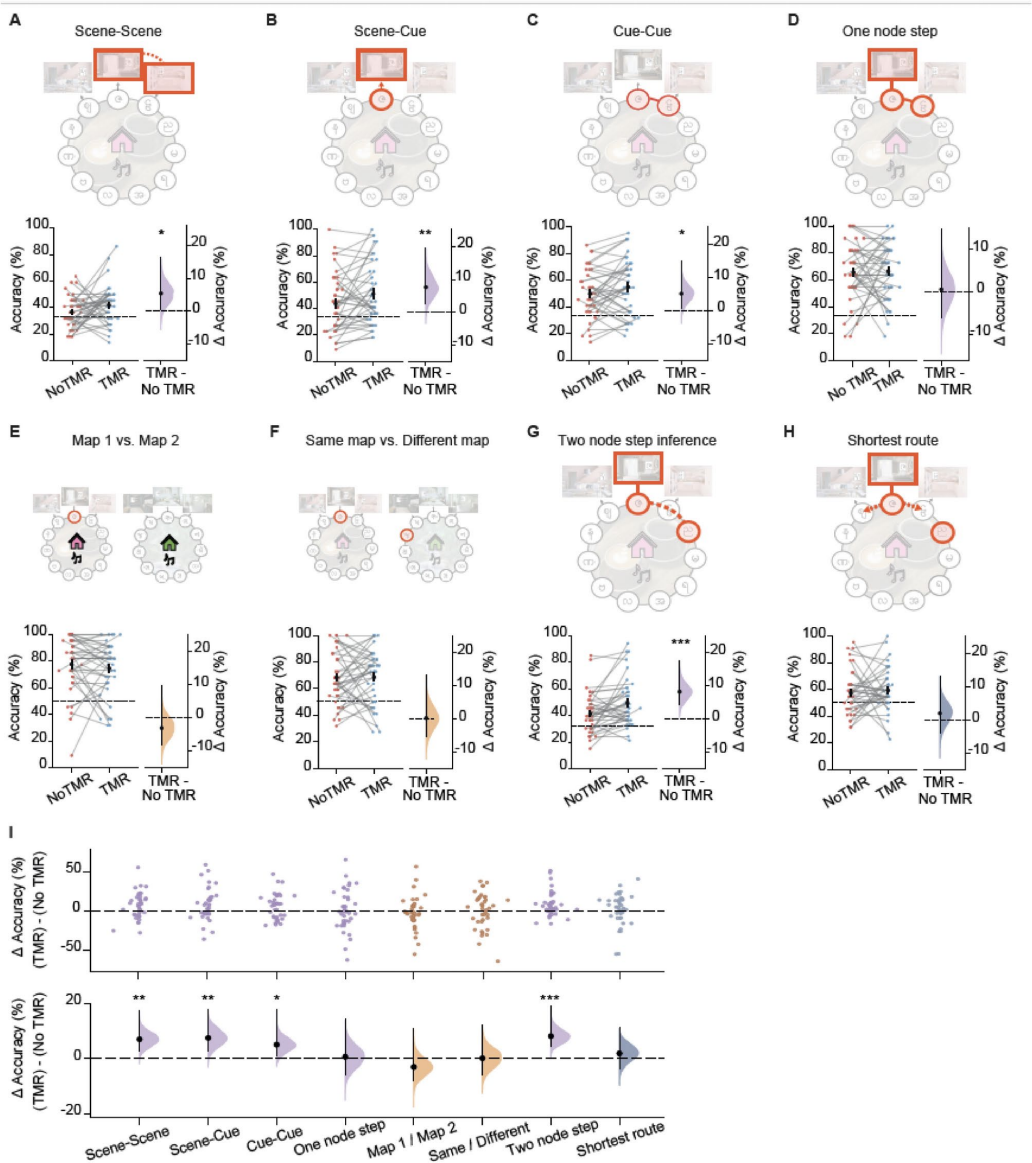
Behavioural performance on each post-rest memory test included in the test phase. **(A-H)** Schematics and results for each of the 8 tests in the order that they were presented to participants, as shown in Fig. 3A: scene-scene, scene-cue, cue-cue, one node step, map1/map2, same/different, two node step inference, and shortest route. Left pair-plots: raw data points for no-TMR map (red; left) and TMR map (blue; right); each data point is mean accuracy for one participant; black dot, participant mean; black ticks ± SEM; black dotted line indicates chance level. Right: difference in mean percentage accuracy between no-TMR and TMR maps shown using bootstrap-coupled estimation (DABEST) plots. Effect size for the difference between no-TMR and TMR groups was computed from 10,000 bias-corrected bootstrapped resamples: black dots, mean; black ticks, one-tailed 95% confidence interval; filled-curve, sampling-error distribution. Purple: associative tests, Orange: contextual tests, Blue-Grey: navigation test. TMR was found to have a significant effect on scene-scene (***A***, p = 0.033), scene-cue (***B***, p = 0.007), cue-cue (***C***, p = 0.029), and two node step inference (***G***, p < 0.001), all of which fall under associative tests. No significant effect was found on one node step (***D***, p = 0.426), map1/map2 (***E***, p = 0.819), same/different (***F***, p = 0.469), or shortest route (***H***, p = 0.284). Notably, these statistical tests were all planned, as outlined in the pre-registration. **(I)** Regression analysis to control for potential confounding variables, as outlined in the pre-registration. Test performance accuracies for the two maps were used as the respective dependent variables, separately for the TMR and no-TMR conditions. Independent variables included the order of the map in training, age, gender, learning accuracy for that map, the colour of the map, and the associated auditory cue. The residuals were obtained for the two models, and the mean added back in, after which the difference in mean percentage accuracy between TMR and no-TMR groups (i.e. the TMR difference) was calculated and plotted. Top: each data point in the swarm plot is mean accuracy for TMR difference for one participant. Bottom: the TMR difference is shown using bootstrap-coupled estimation (DABEST) plots. Effect size for the TMR difference is computed from 10,000 bias-corrected bootstrapped resamples: black dots, mean; black ticks, one-tailed 95% confidence interval; filled-curve, sampling-error distribution. Consistent with the findings reported in Fig. 4A-H, when controlling for potential confounding variables, TMR was still found to have a significant effect on scene-scene (p = 0.001), scene-cue (p = 0.007), cue-cue (p = 0.022), and the two node step inference task (p < 0.001), all of which were categorised as associative tests. Similarly, no significant effect was found on one node step (p = 0.423), map1/map2 (p = 0.168), same/different (p = 0.471), or shortest route (p = 0.255).

We observed no significant effect of TMR on directly trained associations (one node step test, p = 0.426, Figs. 3G, 4D). This result is consistent with previous studies that show no effect of TMR on memory for learned information when TMR is applied during awake rest^21,24–27^. By contrast, for non-directly trained tests, participants performed significantly better on the TMR map (p < 0.001, Fig. 3H). Participants also performed significantly better on the TMR map when considering performance on each of the non-directly trained tests separately, as outlined in the pre-registered analysis (Fig. 4A-C, G). Importantly, these results could not be explained by potential confounding effects, such as differences in learning accuracy, age, gender, order of the map in training, the colour of the map (pink or green), or whether the map was associated with jungle or cafe music (Fig. 4I, Supplementary Fig. 1). These findings suggest that TMR helped participants to identify associations not previously tested (scene-scene, scene-cue), adapt to partial inputs (missing background scenes in cue-cue) and to make novel inferences (two node step). Moreover, when comparing the directly trained one node step with the non-directly trained two node step tests, two tests which were otherwise matched for content and structure, the TMR effect was significantly greater for the non-directly trained two node step test (p < 0.01, Fig. 3I). This suggests that awake, contextual TMR enhances our ability to make novel inferences, compared to recalling trained pairwise associations. Thus, rather than simply strengthening memory for learned associations, TMR-driven memory reactivation facilitates participants’ ability to make novel inferences to effectively extract information that extends beyond direct experience.

### TMR effects are not significantly explained by task difficulty

In a final set of analyses, we asked whether TMR effects are influenced by task difficulty. Notably, to quantify the effect of TMR in any given task, we assess *within-task* measures by comparing performance on the TMR and non-TMR map. These *within-task* measures circumvent the need to control for qualitative differences between tasks which preclude direct comparison of task difficulty. For example, the number of possible choice options varied across tasks, affecting the chance level, as shown in Fig. 4. To quantify the relationship between task difficulty and TMR effect size, we capitalised on across-participant variance *within* each test, and regressed variance in task performance accuracy or reaction time (i.e. two measures of task difficulty) onto the TMR effect size, while controlling for differences in mean performance accuracy or mean reaction time across participants and across tasks. These regression analyses showed no significant effect of performance accuracy or reaction time on TMR effect size (performance accuracy: p = 0.931; reaction time: p = 0.227). In addition, using an exploratory median split analysis we show that high performance accuracy does not appear to mask TMR effects (Supplementary Table 1). Taken together, we observe no significant relationship between task difficulty and TMR effect size. Rather, the differences in TMR effects reported between tasks (e.g. Fig. 3I) are best attributed to differences in the effect of TMR on different types of memory content, with a significant effect of awake, contextual TMR on non-directly trained associative information.

## Discussion

Periods of rest and sleep are reported to facilitate adaptive behaviours, such as our ability to make inferences, perform abstraction and acquire insight^1,2^. However, the mechanism by which these adaptive behaviours emerge remains unclear. Here, using a pre-registered, within-subject, causal manipulation in humans, we investigate whether memory reactivation provides a mechanism for new, adaptive behaviours. We show that memory reactivation during rest improves our ability to make novel associative inferences, with no effect on recall for learned information. These findings suggest that memory reactivation during rest provides a mechanism to support new, adaptive behaviours that draw from unobserved information.

To demonstrate the effect of memory reactivation on human behaviour we designed a TMR manipulation to causally bias the content of memory reactivation. We apply this TMR manipulation to a rich behavioural paradigm that provides an opportunity to quantify the effect of TMR on different aspects of memory. As predicted in our pre-registration, we show that TMR facilitates average performance accuracy across all tests. However, when considering the effect of TMR on different aspects of memory, we show that TMR improves memory performance only for associations that have not been directly learned, with no effect on directly trained associations, contextual memory or navigation tests. Therefore, our findings suggest that rather than simply strengthening memory for learned associations, memory reactivation during awake rest plays a critical role in generating new links that draw on implicit or indirect experience to support adaptive behaviour. For example, constructing the two step link ‘A’- ‘B’- ‘C’ when only ‘A’- ‘B’ and ‘B’- ‘C’ have been experienced.

The benefit of TMR for non-directly trained associations reported here is congruent with evidence to suggest memory reactivation during awake rest prioritizes weakly learned information^28^. Moreover, previous studies show that application of TMR in the awake state benefits associative memory stability for weak associations, whereas strongly learned associations are not reliably influenced by re-exposure^29^. Our study builds on this previous work by testing the effect of TMR during awake rest on directly and non-directly trained associations. In demonstrating an effect of TMR on non-directly trained associations, our findings suggest that memory reactivation prioritises formation of associative links that extend beyond direct experience. Non-directly trained associations may therefore share physiological properties with weaker memories, rendering them susceptible to TMR.

However, the significant differences in TMR effect between different tasks in the test phase cannot be merely explained by differences in task difficulty. We note that our *within-task* measures of the TMR effect size control for qualitative differences between tasks. For example, across tasks in the test phase there was variation in the number of options at choice, which affected the chance level (indicated in Fig. 4). Importantly, for the two contextual tasks and for the navigation task, the chance level was higher (50%), and, correspondingly, the average performance accuracy was higher than for other tasks where chance was set to 33%. Using multivariate regression and median split analyses to investigate the effect of task difficulty on TMR effect size, we show no evidence for a significant relationship between the TMR effect size and either performance accuracy or reaction time. Thus, rather than task difficulty accounting for differences in the reported TMR effects, we conclude that different aspects of memory are differentially susceptible to TMR.

We note that the benefit of TMR during awake rest reported here is only observed for *associations* that have not been directly trained, including the two-node step inference test. When testing participants’ ability to navigate around the maps (shortest route), no effect of TMR was observed. Interestingly, both the two-node step inference test and the navigation test required participants to assess the relationship between nodes positioned two steps apart. However, while in the two node step test participants were instructed to infer the cue located two nodes away, for the navigation test participants were given the cue located two nodes away and were instructed to infer the shortest route towards that cue, by indicating a left or rightwards trajectory from the current node. Circumstantial evidence suggests participants adopted a different strategy in these two different tests: participants’ reaction times on the navigation test were significantly faster than that observed on the two-node step inference test (p < 0.001). Thus, a tentative explanation is that participants relied on ‘gist’ memory to solve the navigation test, drawing on memory for general features of the maps, rather than memory for singular nodes which may guide performance on the two node step inference test.

Gist memory may be considered to fall on a continuum between episodic and semantic memory, with increasing levels of abstraction^30^. Compared to episodic-like memory, gist memory appears to be supported by partially distinct mechanisms, which bear different relationships to memory reactivation. Previous studies suggest that gist memory for non-directly trained abstraction may be susceptible to TMR when applied during periods of sleep with high spindle density^31^. However, when the effects of sleep on episodic-like memory and gist memory are directly compared, sleep immediately after encoding is found to promote consolidation of episodic-like memory recall 10 h later, while abstract gist only benefits at one year^32^. Performance on the navigation task may therefore only be susceptible to TMR across longer timescales or after sleep with high spindle density. Thus, the awake contextual TMR protocol presented here shows no benefit on participants’ ability to plan efficient and directed trajectories around the map. Instead, awake contextual TMR appears to prioritise the formation of links that go beyond direct experience, to benefit associative inference.

Our study builds on physiological data demonstrating that *offline *periods of rest and sleep are characterised by high frequency oscillations in the local field potential, described as Sharp-Wave Ripples (SWRs)^8–10^. Spiking sequences that occur during SWRs play back encoded information, a phenomenon known as replay^8–10^. For example, in the sleeping rodent brain, hippocampal spiking sequences resemble virtual trajectories through the animals’ learned environment^33^. This ‘replay’ is thought to account for the memory benefits of offline periods^34,35, ^as the timescale is concomitant with synaptic plasticity^36^. However, replay does not always mirror waking experience. Hippocampal spiking sequences are also reported to reflect novel trajectories through the animals’ environment^15–17^, and co-activation between cues that have never been directly paired^6^. Therefore, rather than merely strengthening and consolidating learned information, offline memory reactivation may provide a mechanism to edit and build mnemonic codes that deviate from veridical experience. Our study supports this hypothesis by demonstrating that new, adaptive human behaviours emerge when memory reactivation is manipulated using TMR during awake rest.

Our TMR protocol differs from those used in previous studies in two key ways. First, rather than just quantifying the effect of TMR on learned information, we use a rich behavioural paradigm to assess the potential diverse effects of memory reactivation on behaviour. Thus, we can directly compare the effect of TMR on different aspects of memory, showing that memory reactivation during awake rest improves performance on new behaviours, such as associative inference, with no effect on behaviours that draw directly from learned information. Second, in contrast to the majority of previous TMR studies, we use contextual rather than punctate auditory cues to trigger memory reactivation. Thus, our TMR protocol, although applied in awake rest, employs a TMR protocol that is arguably more comparable to TMR studies that have used contextual odor cues in sleep^24,37,38^. By using a contextual cue, we bias the content of memory reactivation towards an entire memory map, rather than a specific cue or event. The aim of this approach is to capitalise on the endogenous neural dynamics of reactivation within each memory map, without interrupting or altering the natural sequence of replay, as may occur with the introduction of punctate cues.

These aspects of the TMR protocol must be considered when comparing our reported effect of TMR with that of others. Previous studies demonstrate that TMR during periods of post-encoding sleep elicits a robust effect on memory retention^21,24,25,39–45^, although nuanced TMR protocols applied during sleep have been used to elicit forgetting^46,47^. Notably, more variable results are reported when using TMR during periods of awake rest^21,24–27,29,39,48,49^, which may in part be explained by variation in the level of task engagement during the rest period^29,39^. Specifically, application of TMR in the awake state appears to show no benefit for memory when cueing is coupled with externally-orientated, attention demanding tasks^21,39^, while cueing during a state of reduced external engagement appears to benefit weak memories^29^. Our results provide further clarity on the effect of TMR in awake rest, in demonstrating that TMR during awake rest improves performance for non-directly trained information. Moreover, in contrast to effects of TMR reported in sleep, there is no significant effect of awake TMR on memory retention for learned information. This highlights potential differences in the demands of wakeful rest and sleep, despite both states playing a pivotal role for stabilising new learning^35,50–54^.

To incentivise participants, we included monetary reward in both the learning and test phase. There is clear evidence to suggest reward can bias the content of memory^55–57^, and interact with the effect of TMR^39^. Here, we did not differentially manipulate reward across the two maps. Indeed, we controlled for any effect of monetary reward on TMR by providing monetary bonus for performance across both maps. In addition, the bonus was only indicated at the end of a learning or test session, to prevent any relationship between reward and either of the two maps. Consequently, any effect of monetary reward on TMR should be equivalent across both maps, and cannot explain the reported *between-map* TMR effects.

Importantly, perturbations in memory reactivation are emerging as a core physiological marker of neuropsychiatric disease. For example, replay is reduced in Alzheimers’ model mice^58,59^ and memory reactivation is disrupted in patients with schizophrenia^60^. Unlike the majority of auditory TMR protocols that deliver punctate cues during periods of sleep, here we deliver TMR during awake rest using a contextual auditory manipulation. This simple, low-tech, low-cost solution for manipulating memory reactivation carries important translational potential, and can be readily embedded in wearable technologies as a therapeutic tool at home, in educational or in rehabilitation settings^43,61^.

Overall, our findings demonstrate that applying contextual TMR during awake rest provides a tool to strengthen the formation of novel associative links between sensory cues. By contrast, no effect of TMR was observed on directly learned associations. In addition, no effect of TMR was observed on contextual memory or navigation tests that both rely on map-level knowledge. Taken together, these findings suggest that memory reactivation during wakeful rest provides a mechanism to generate previously untrained associations, to support new adaptive behaviours such as inference.

## Methods

### Pre-registration

Prior to starting data collection or analyses, this experiment was pre‐registered on Open Science Framework (OSF) REGISTERIES using a pre-registration template. The pre‐registered report is located at^62^.

### Participants

40 healthy volunteers participated in the study (mean age of 24.3 ± 3.28 years, 13 males). All participants had normal or corrected-to-normal vision. All participants gave informed written consent. 10 participants were excluded with replacement due to failure to meet data quality checks specified in our pre-registration, namely: meeting the learning criterion (see below); familiarity with written Hindi or Burmese; non-compliance with task instructions, such as turning off the auditory cue. This study was approved by the University of Oxford ethics committee (reference number: R43594/RE013). All methods were performed in accordance with the relevant guidelines and regulations.

### Experimental set-up

All behavioural tasks were coded in MATLAB (version R2021b) using Psychophysics Toolbox-3 (version 3.0.18)^63^. The tasks were presented on a MacBook Pro (M2, 2022) with a 13-inch screen and spatial resolution of 2560 × 1600pixels (width x height). All behavioural tasks were performed within a single day and study visit.

### Learning phase

In the first stage of the study (‘learning phase’, Fig. 1A), participants learned two maps of associations. Each map consisted of 11 rooms, or nodes, in a consistent colour scheme – green or pink (Fig. 1B-C). Rooms consisted of a unique background scene paired with a cue (symbol). Cues were letters from the Hindi and Burmese alphabets (Fig. 1). The cue was positioned within a picture frame situated in a plausible location within the scene (i.e. on the wall). The order of scenes, allocation of cues to maps and scene-cue pairings were randomised across participants. Therefore, there were not any consistent/systematic relationships between neighbouring nodes in a map, including the spatial relationships between cues. Scene-cue pairs (rooms) constituted nodes in the ring-structured maps, which participants navigated in a clockwise direction only (Fig. 1B-C). The ring structure was never made explicit to participants.

During learning, each map was paired with a background soundtrack. This background soundtrack provided a contextual cue that could subsequently be used to bias memory reactivation for one of the two maps using TMR^18–21^. Thus, while learning one map, participants heard a cafe soundtrack (piano music and cafe sounds), while for the other map they heard a jungle soundtrack (flute music and jungle sounds). Across participants, the following conditions were fully counterbalanced: pink/green house stimuli, cafe/jungle music, order in which maps were learned across learning blocks, which map was used for TMR. Overall, this created 8 fully counterbalanced conditions which were assigned to participants in a pseudo-randomised manner, with 5 repeats per counterbalanced condition across participants.

During the learning task, participants learned room-to-cue associations using three-alternative forced choice. On each trial of the learning task, a probe cue was presented embedded in its corresponding scene (e.g., probe cue within pink living room, Fig. 1D), while three choice cues were presented in randomised positions across the bottom of the screen. The choice cues included the cue from the next room in the house (clockwise direction), together with two other cues from the same house to which the probe was not associated. The cue from the previous room in the house (anticlockwise direction) was never presented as an option. Participants were given up to 10 s to select the cue from the next room in the house using the keyboard (‘b’, ‘n’, ‘m’ keys to select between left, middle, and right options). If participants picked the correct cue, it was highlighted by a green box. If participants picked an incorrect symbol it was highlighted by a red box. Regardless of whether they made a correct or incorrect choice, after 1 s of feedback, the next room in the map (clockwise direction) together with its cue were presented as a probe for the next trial. Participants completed sequences of four trials travelling around the circle before moving to a new random start location. The relevant soundtrack for each map played throughout all trials. For example, cafe music played throughout all pink house trials and jungle music played throughout all green house trials.

In the first learning session, participants performed four mini-blocks of the task in total, alternating between blocks of trials on each of the two maps (Fig. 1E). Across all four mini-blocks, participants completed one sequence starting from each of the 11 nodes in each map. The first two mini-blocks contained 5 sequences and the final two mini-blocks contained the remaining 6 sequences (Fig. 1E). As above, the order in which the two maps were presented (including whether it was green or pink and whether it was later used for TMR) was counterbalanced across participants.

Before starting the learning task, participants were told that they would receive a £2 reward if they achieved a criterion of at least 75% accuracy, across both maps. In the first learning session, participants received feedback on their overall accuracy for both maps at the end of the four mini-blocks (two mini-blocks for each map). If participants failed to reach 75% accuracy at the end of the first learning session then they were given additional training. Across the first learning session, participants achieved a mean accuracy of 69.77% (SEM = 1.93%) (Fig. 1E). Of the 40 participants, 2 reached the 75% criterion at this stage, and the remaining 38 went on to complete additional training in the second learning session.

The second learning session consisted of a maximum of 16 additional training blocks of the learning task (Fig. 1F)*.* As in the first learning session, the relevant contextual soundtrack for each map was played throughout. Each block tested one complete path around a map (clockwise direction), broken into two sub-sequences of 5 or 6 trials. Participants received feedback at the end of each pair of blocks and exited the task when they achieved > 75% accuracy across both maps. Participants who failed to reach a criterion accuracy of 75% across both maps after 16 blocks were excluded from the remainder of the study and compensated for their time. Four subjects failed to reach criterion and were excluded with replacement. Their data is not included in any figures or analysis. Those participants that did reach criterion (> 75% accuracy across both maps) were informed of their £2 bonus, and progressed to the next stage of the experiment.

### *Rest + TMR manipulation*

After the learning task, participants underwent a period of awake rest which was combined with a TMR manipulation (rest + TMR), lasting 90 min in total (Fig. 1A). During the rest + TMR period, participants were instructed to alternate between 10 min of eyes-closed awake-rest and 20 min of completing a jigsaw puzzle, which they repeated three times over. During the rest + TMR period, the contextual soundtrack associated with one of the two maps was played (i.e., either café or jungle music associated with either the pink or the green map). To encourage attention towards the background track, the music was played in an intermittent fashion, being turned on and off every 12 s on average. The length of both ‘on’ and ‘off’ periods were randomly drawn from truncated gamma distributions with a minimum of 9 s, maximum of 16 s and mean of 12 s. As noted above, the contextual soundtrack used for TMR, it’s pairing with the green or pink map and the order of learning, was fully counterbalanced across participants.

### Test phase

After the rest period and accompanying TMR manipulation, participants commenced the ‘test phase’. During the test phase, we assessed participants’ ability to recall information learned during the learning phase. We also assessed participants’ ability to make novel inferences. To this end, we designed eight different tests to investigate different aspects of memory. Namely, we assessed how well participants could: recall associative information (i.e. *within-map* associations) for both directly and non-directly trained associations; recall contextual information (i.e. ability to delineate *between* the two maps); navigate around the underlying maps. Each of the eight different tests are described in more detail below, in the order in which they were presented to participants. Labels are included to indicate which aspects of memory the test was designed for:

1. Scene-Scene: scene-scene associations across neighbouring nodes within each map (**associative, non-directly trained**; Fig. 2A)
2. Scene-Cue: scene-cue association within each node, within each map (**associative, non-directly trained**; Fig. 2B)
3. Cue-Cue: cue-cue associations across neighbouring nodes within each map (**associative, non-directly trained**; Fig. 2C)
4. One node step: room (scene + cue) to cue pairwise transitions (**associative, directly trained**; Fig. 2D)
5. Map 1/Map 2: whether each cue belongs to map 1 or map 2 (**contextual**; Fig. 2E)
6. Same/Different: whether each possible pair of cues belong to the same or different map (**contextual**; Fig. 2F)
7. Two node step: scene + cue to cue transitions across two node steps (**associative, non-directly trained**; Fig. 2G)
8. Shortest route: whether the shortest route from a starting node (scene + cue) to a target cue is in the clockwise or anti-clockwise direction. The target cue was always two steps away from the starting node (**navigation**; Fig. 2H)

The ‘one node step’ had the same trial content and structure as the three-alternative forced choice trials included in the learning task, and was thus classified as *directly trained*. The trial order was shuffled to test knowledge of node (scene + cue) to cue associations in the absence of extended trajectories around the map.

Notably, at pre-registration, ‘cue-cue’ (test 3) was classified as directly trained. However, as the background scenes were not included in this test the trial content and structure differed from trials included in the learning task. The ‘cue-cue’ test was therefore classified as non-directly trained for all data reported in the *Results* section. For consistency with the pre-registration, we nevertheless note that the reported TMR effects for directly and non-directly trained tests remained when the performance on the ‘cue-cue’ test was classified as directly trained, as in the pre-registration (TMR vs. no-TMR: directly trained: p = 0.111; non-directly trained: p < 0.001).

Importantly, the background soundtracks (café or jungle music) were not played during the test phase. Three-alternative forced choice was used in tests (1), (2), (3), (4), and (7), where participants could pick between three options presented at the bottom of the screen using the keyboard (‘b’, ‘n’, ‘m’ keys to select between left, middle, and right options). A two-alternative forced choice task was used in tests (4), (5) and (8), where participants could pick between two options using the keyboard (‘b’ or ‘m’ keys to select between left and right options). The chance level therefore varied across tasks, as indicated in Fig. 4. In the two node step test, the stimulus was presented on screen for 6 s, before participants were given 1.5 s to make their choice. In all other tasks, each stimulus was presented on screen for up to 10 s, during which participants were required to respond or were otherwise moved on to the next stimulus without logging a response (with no inter-trial intervals). All chosen options were highlighted by a yellow box, regardless of accuracy. Participants received feedback on their overall performance at the end of all tests and received a bonus reward of £3 if they scored 50% or above across all tests.

### Statistical analysis

Statistical analysis was performed in MATLAB (version R2021b) and in Python v.3.9. The Python packages DABEST^64^(data analysis with bootstrap-coupled estimation), numpy^65^, matplotlib^66^, scipy^67^, seaborn^68^and pandas^69^ were used to analyse data and generate plots. Figures were made using Adobe Illustrator v.27.3. Analysis for each of the eight final tests was planned and pre-registered.

As specified in the pre-registration, using data from the test phase, we assessed evidence for a within-subject TMR effect on test accuracy using DABEST plots with bootstrap estimation^64^ (pre-registered analyses: CI = 95%, one-tailed; exploratory analyses: CI = 95%, two-tailed). As specified in the pre-registration, we also assessed reaction times (Supplementary Figs. 2–3). To control for potential confounding sources of variance, we performed multivariate regression using performance across all tasks, using separate models for the TMR and the no-TMR maps, with independent variables as follows: the order of map in training, age, gender, learning phase accuracy for the relevant map, green or pink, and jungle or café music. The residuals were obtained from both models (one for each map), following which the mean was added back in, and the difference between TMR and no-TMR maps was obtained for DABEST analysis (Fig. 4I). To investigate the relationship between TMR effect size and task difficulty we performed multivariate regression, with the dependent variable set to the TMR effect size for each task, for each participant, for each map, and the independent variables as follows: either mean performance accuracy or mean reaction time; task number; participant number.

## Supplementary Information

Supplementary Information (PDF 1664KB).

## Data Availability

Upon publication the data and code used in this study will be made available via the MRC BNDU Data Sharing Platform (https://data.mrc.ox.ac.uk) at the following DOI 10.60964/bndu-wv4h-0734.
