# Author Correction: Memory reactivation generates new, adaptive behaviours that reach beyond direct experience

**DOI:** 10.1038/s41598-025-89146-2

**Published:** 2025-02-10

**Authors:** Annalise B. Rawson, Sumedha Nalluru, Jill X. O’Reilly, Helen C. Barron

**Affiliations:** 1 Wellcome Centre for Integrative Neuroimaging, University of Oxford, FMRIB, John Radcliffe Hospital, Oxford, UK; 2 Medical Research Council Brain Network Dynamics Unit, Nuffield Department for Clinical Neurosciences, University of Oxford, Oxford, UK; 3 Department of Experimental Psychology, University of Oxford, Oxford, UK

Correction to: *Scientific Reports* 10.1038/s41598-024-78906-1, published online 03 December 2024

The original version of the Article contained an error in the Author Information section.

“Annalise B. Rawson, Sumedha Nalluru, Jill X. O’Reilly and Helen C. Barron contributed equally to this work.”

now reads:

“Annalise B. Rawson and Sumedha Nalluru contributed equally to this work.

Jill X. O’Reilly and Helen C. Barron jointly supervised this work.”

The original Article has been corrected.

