# Supplementary material for: Memory reactivation generates new, adaptive behaviours that reach beyond direct experience
Source: Sci Rep. Author manuscript; Available in PMC 2024 Dec 6. (PMC11615380; doi:10.1038/s41598-024-78906-1)
Supplement: Supplementary Material [file EMS201159-supplement-Supplementary_Material.pdf]

Supplementary Materials

Supplementary Tables

	Test	TMR effect size (mean difference, %)	Bootstrapped p-value	Low or high split	Split mean (%)
**Grouped tests with TMR effect**	**All tests**	2.00	0.025	Low	49.99
		4.56	0.002	High	63.42
	**Associative tests**	3.85	0.000	Low	42.64
		6.97	0.000	High	57.61
	**Non-directly trained tests**	4.41	0.000	Low	37.43
		8.77	0.000	High	54.86
**Individual tests with TMR effect**	**Scene-Scene**	5.23	0.035	Low	32.39
		5.68	0.035	High	46.25
	**Scene-Cue**	7.73	0.000	Low	31.14
		7.50	0.028	High	63.75
	**Cue-Cue**	0.00	0.504	Low	38.41
		10.23	0.000	High	66.48
	**Two node step**	4.24	0.006	Low	35.45
		12.12	0.000	High	55.30
**Grouped tests with no TMR effect**	**Contextual tests**	-2.84	0.829	Low	58.81
		0.11	0.477	High	85.85

**Individual tests with no TMR effect**	**Map 1/Map 2**	-3.64	0.806	Low	62.05
		-2.27	0.881	High	90.23
	**Same/Different**	-2.27	0.708	Low	54.77
		2.73	0.181	High	82.27
	**One node step *(Directly trained)***	-1.36	0.602	Low	52.50
		2.73	0.174	High	79.55
	**Shortest route *(Navigation)***	3.41	0.151	Low	49.43
		0.45	0.455	High	67.27

Supplementary Table 1 | Exploratory post-hoc median split analyses to investigate the relationship between task difficulty and TMR effect size

To address a reviewer's comment about a possible relationship between task difficulty and TMR effects, we performed an exploratory post-hoc median split analysis. Note, across these exploratory analyses we did not correct for multiple comparisons. For each task in the test phase we divide participants into high and low performing participants, by applying a median split to overall performance accuracy for the test(s) in question. For both low and high performing participants, we then assess the difference in performance accuracy between the TMR and no-TMR maps. Results are included for both individual tests and for tests grouped according to memory type (according to the analyses presented in Figure 3). Bootstrapped p-values were obtained through 10,000 bootstrapped resamples of the differences in accuracy between TMR and no-TMR maps. For all tests where a significant TMR effect was observed across participants (*Figure 3-4*), the effect was still observed (and typically larger) in high performing participants. Therefore, rather than TMR effects being masked by high performance accuracy on easier tasks, these results suggest that TMR effects remain detectable when participants find a test easier. Consistent with this interpretation of the data, for all tests where no significant TMR effect was observed across participants (*Figure 3-4*), we continue to see no significant TMR effect in both high and low performing participants. Together, these exploratory analyses suggest that differences in the TMR effect between tasks cannot be explained by task difficulty but instead reflect differential sensitivity of task content to TMR. These conclusions are corroborated by regression analyses reported in the *Results* section showing no significant effect of task difficulty (measured using performance accuracy or reaction time) on TMR effect size.

Supplementary Figures

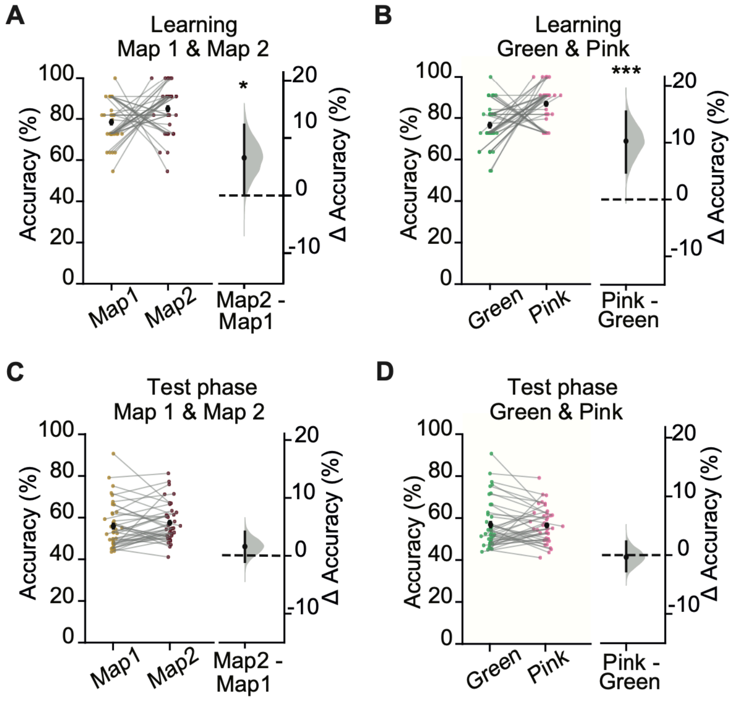

Supplementary Figure 1 | Effect of map order and colour on performance accuracy

(A-D) Left: raw data points showing performance accuracy by map, when the two maps are grouped either by the order presented in learning (map 1 (mustard) vs. map 2 (maroon), where map 1 is presented first in learning; ***A,C***), or by map colour (green vs. pink, ***B,D***). Despite qualitatively matching the node content across maps, in the final learning block we observed a significant difference in performance accuracy between map 1 and map 2 (map2>map1, p=0.046, ***A***) and between the green and pink maps (pink>green, p<0.001, ***B***). However, during the testing phase we observed no significant difference between map 1 and map 2 (map1 vs. map2: p=0.258, ***C***), or between the green and pink maps (green vs. pink: p=0.804, ***D***). Importantly, across participants we fully counterbalanced the colour of the map, the order of map presentation and the contextual auditory cues. Therefore, differences in learning accuracy due to the colour or order of the map presentation cannot explain the TMR effects we report. The regression analysis we show in *Figure 4I* further demonstrates this point, where all reported significant TMR effects are retained when controlling for potential confounding variables that include map colour and map order. Each data point is mean accuracy for one participant; black dot, mean; black ticks ± SEM. Right: difference in mean percentage accuracy between the two maps shown using bootstrap-coupled estimation (DABEST) plots. Effect size for the difference between the two maps was computed from 10,000 bias-corrected bootstrapped resamples: black dots, mean; black ticks, two-tailed 95% confidence interval; filled-curve, sampling-error distribution.

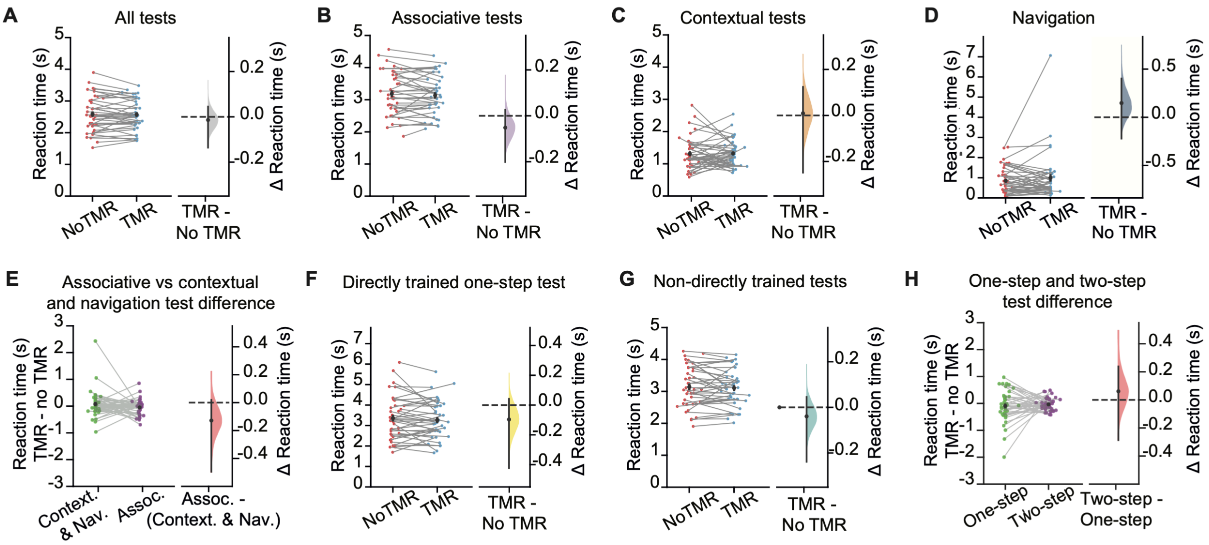

Supplementary Figure 2 | Reaction time data on post-rest memory tests, grouped by memory content, relating to Figure 3

Difference in reaction times between the TMR and no-TMR maps on final tests, grouped according to memory type. **(A-H)** Left: raw data points for no-TMR group (red; left) and TMR group (blue; right) **(*A-D, F-G*)**, or TMR difference in reaction time for two different types of task (green, left; purple, right) **(*E, H*).** Each data point is mean reaction time ***(A-D, F-G)*** or mean TMR difference in reaction time ***(E-H)*** for one participant; black dot, mean; black ticks ± SEM. Right: difference in mean reaction time between no-TMR and TMR groups **(*A-D, F-G*)** or difference in mean reaction time between no-TMR and TMR groups between two different types of task **(*E,H*)**, shown using bootstrap-coupled estimation (DABEST) plots. Effect size for the difference between no-TMR and TMR groups was computed from 10,000 bias-corrected bootstrapped resamples of the differences in reaction time between TMR and no-TMR maps: black dots, mean; black ticks, one-tailed 95% confidence interval; filled-curve, sampling-error distribution. No significant effect of TMR was observed in reaction time data across all tests (***A***, p=0.346), on associative tests (***B***, p=0.133), on contextual tests (***C***, p=0.549), on navigation tests (***D***, p=0.855), when comparing associative and contextual/navigation tests (***E***, p=0.089), on the directly trained one node step test (***F***, p=0.148), on non-directly trained tests (***G***, p=0.225), or when comparing indirect two node step test with direct one node step test (***H***, p=0.706). Unfortunately, due to an error in the code, during data acquisition the reaction times for the same/different test were over-written and we are unable to report this result. Therefore, the contextual tests group contains map1/map2 only. Notably, these statistical tests were all planned, as outlined in the pre-registration.

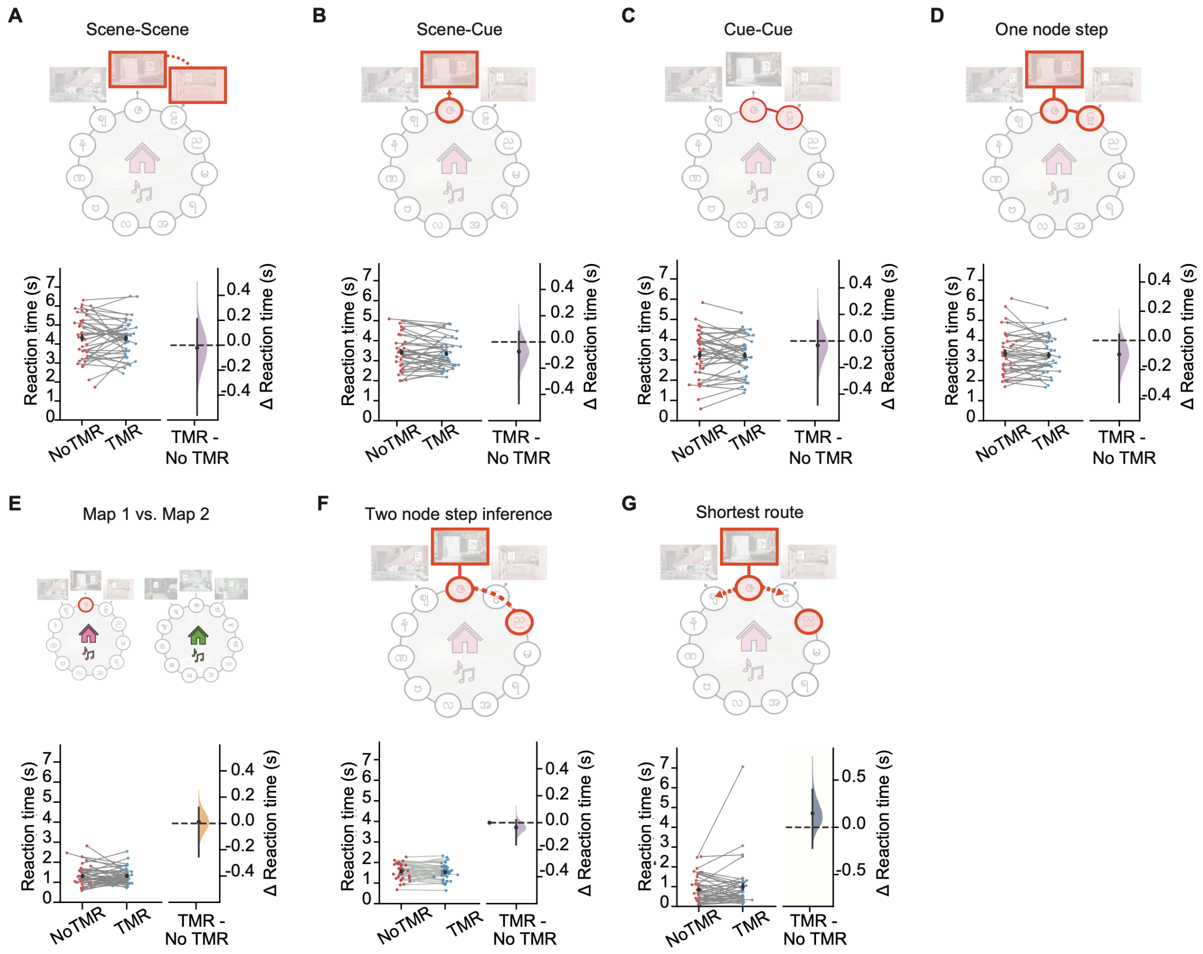

Supplementary Figure 3 | Reaction time data for each task in the test phase, relating to Figure 4

Difference in reaction times between the TMR and no-TMR maps for each of the tests included in the test phase. **(A-G)** Schematics and results for each of the 8 tests in the order that they were presented to participants, as shown in Figure 3A: scene-scene, scene-cue, cue-cue, one node step, map1/map2, two node step inference, and shortest route. Left pair-plots: raw data points for no-TMR map (red; left) and TMR map (blue; right); each data point is mean reaction time for one participant; black dot, participant mean; black ticks ± SEM. Right: difference in mean reaction time between no-TMR and TMR maps shown using bootstrap-coupled estimation (DABEST) plots. Effect size for the difference between no-TMR and TMR groups was computed from 10,000 bias-corrected bootstrapped resamples: black dots, mean; black ticks, one-tailed 95% confidence interval; filled-curve, sampling-error distribution. Purple: associative tests, Orange: contextual tests, Blue-Grey: navigation test. No significant effect of TMR was observed in reaction time data from scene-scene (***A***, p=0.441), scene-cue (***B***, p=0.210), cue-cue (***C***, p=0.387), one node step (***D***, p=0.148), map1/map2 (***E***, p=0.549), two node step inference (***F***, p=0.171), or shortest route (***G***, p=0.855). Unfortunately, due to an error in the code, during data acquisition the reaction times for the same/different test were over-written and we are unable to report this result. Notably, these statistical tests were all planned, as outlined in the pre-registration.
